# Supplementary material for: Macrophages in Oral Carcinomas: Relationship with Cancer Stem Cell Markers and PD-L1 Expression
Source: Cancers (Basel). 2020 Jul 2;12(7):1764. doi: 10.3390/cancers12071764 (PMC7408350; doi:10.3390/cancers12071764)
Supplement: Supplementary file 1 [file cancers-12-01764-s001.pdf]

Supplementary Materials

Macrophages in Oral Carcinomas: Relationship with Cancer Stem Cell Markers and PD-L1 Expression

Faustino J. Suárez-Sánchez, Paloma Lequerica-Fernández, Julián Suárez-Canto, Juan P. Rodrigo, Tania Rodriguez-Santamarta, Francisco Domínguez-Iglesias, Juana M. García-Pedrero and Juan C. de Vicente

Table S1. Univariate disease-specific survival analysis according to CD68 expression in 121 OSCC patients.

CD68 Percentile	No. Cases	Censored Patients (%)	Disease-Specific Survival (95% CI)	*p*
Stromal CD68				
< P25	30	16 (53)	110.50 (80.02–140.97)	0.68
≥ P25	95	56 (59)	135.15 (112.95–157.34)	
Tumoral CD68				
< P25	31	15 (48)	117.17 (82.60–151.73)	0.48
≥ P25	94	57 (61)	139.06 (116.49–161.64)	
Stromal CD68				
< P50	63	36 (57)	134.67 (108.04–161.30)	0.88
≥ P50	62	36 (58)	129.00 (102.98–155.03)	
Tumoral CD68				
< P50	63	31 (49)	117.89 (91.90–143.88)	0.12
≥ P50	62	41 (66)	125.25 (105.02–145.48)	
Stromal CD68				
< P75	94	57 (61)	139.77 (117.03–162.50)	0.18
≥ P75	31	15 (48)	94.65 (67.23–122.07)	
Tumoral CD68				
< P75	93	50 (54)	125.12 (102.80–147.44)	0.19
≥ P75	32	22 (69)	129.73 (102.30–157.15)	

Table S2. Univariate disease-specific survival analysis according to CD163 expression in 121 OSCC patients.

CD163 Percentile	No. Cases	Censored Patients (%)	Disease-Specific Survival (95% CI)	*p*
Stromal CD163				
< P25	31	15 (48)	101.64 (71.20–132.08)	0.13
≥ P25	90	55 (61)	142.31 (120.14–164.48)	
Tumoral CD163				
< P25	29	15 (52)	126.47 (91.23–161.72)	0.73
≥ P25	92	55 (60)	138.55 (115.94–161.16)	
Stromal CD163				
< P50	60	32 (53)	110.95 (89.28–132.62)	0.29
≥ P50	61	38 (62)	142.93 (116.16–169.71)	
Tumoral CD163				
< P50	61	36 (59)	138.64 (111.79–165.49)	0.71
≥ P50	60	34 (57)	105.36 (86.33–124.38)	
Stromal CD163				
< P75	89	50 (56)	134.57 (112.24–156.89)	0.34
≥ P75	32	20 (62)	144.42 (112.05–176.79)	
Tumoral CD163				
< P75	92	50 (54)	128.50 (106.53–150.47)	0.31
≥ P75	29	20 (69)	122.14 (95.17–149.12)	

p values were estimated using the log-rank test. 95% CI: 95% Confidence Interval.

© 2020 by the authors. Licensee MDPI, Basel, Switzerland. This article is an open access article distributed under the terms and conditions of the Creative Commons Attribution (CC BY) license (http://creativecommons.org/licenses/by/4.0/).
